# Dissociation as a causal pathway from sexual abuse to positive symptoms in the spectrum of psychotic disorders

**DOI:** 10.1186/s12888-021-03290-3

**Published:** 2021-05-24

**Authors:** Mohsen Khosravi, Nour-Mohammad Bakhshani, Niloofar Kamangar

**Affiliations:** 1 Department of Psychiatry and Clinical Psychology, Zahedan University of Medical Sciences, Zahedan, Iran; 2 Department of Psychology, Children and Adolescent Health Research Center, Zahedan University of Medical Sciences, Zahedan, Iran; 3 General Practitioner, Zahedan University of Medical Sciences, Zahedan, Iran

**Keywords:** Dissociation, Sexual abuse, Psychotic disorders

## Abstract

**Background:**

Although numerous studies have supported the role of childhood maltreatment in the etiology of psychosis, underlying mechanisms have not been well understood yet. The present study aimed to investigate the mediating role of particular forms of dissociation in the relationship between five major types of childhood abuse and psychotic symptoms among patients with schizophrenia spectrum and other psychotic disorders.

**Methods:**

In this cross-sectional correlation study, 70 first-episode psychotic patients and 70 chronic psychotic patients were selected by systematic random sampling (with the sampling interval of 3) from among inpatients and outpatients referring to Baharan Psychiatric hospital, Zahedan, Iran, and were matched based on age, gender, and education level. Moreover, 70 age-, gender-, and education level-matched community controls were recruited from hospital staff and their relatives and friends. All of the participants completed a research interview and questionnaires. Data on experiences of childhood maltreatment, psychosis, dissociation, and demographics were collected and analyzed by SPSS V25 software.

**Results:**

The obtained results revealed that the mean scores of sexual abuse, emotional abuse, and physical abuse were higher in psychotic patients than community controls (without any significant difference between first-episode psychotic patients and chronic psychotic patients). Furthermore, the highest mean scores of dissociative experiences belonged to chronic psychotic patients. Multiple-mediation also indicated that absorption and dissociative amnesia played a mediating role in the relationship between sexual abuse and positive symptoms. Moreover, this study demonstrated the role of physical abuse in predicting psychotic symptoms even in the absence of sexual abuse.

**Conclusions:**

This study illustrated specific associations among childhood maltreatment, dissociative experiences, and psychotic symptoms in the clinical population. Thus, to provide appropriate interventions, patients with schizophrenia spectrum and other psychotic disorders were asked about a wide range of possible adverse childhood experiences and dissociative experiences. Nevertheless, further studies using prospective or longitudinal designs need to be carried out to realize the differential contribution of various forms of childhood maltreatment and their potential interactions, more precisely.

## Background

Childhood adversities have been recognized as a known risk factor in numerous mental disorders [1, 2]. As recently recommended, childhood maltreatment such as sexual abuse, physical abuse, emotional abuse, and neglect, might be accompanied by an increase in psychosis risk [3–7]. In this respect, a recent meta-analysis of 29 studies showed that the severity of the positive and negative symptoms was associated with childhood sexual abuse and neglect, respectively. However, no association was observed between positive symptoms severity and childhood neglect and between negative symptoms severity and childhood sexual abuse [7].

In recent decades, the long-term negative effects of childhood maltreatment on the social and cognitive functioning of psychotic patients have led to significant researches into the understanding of possible underlying psychological mechanisms [8–13]. In this regard, numerous psychological models have been proposed to explain the association between childhood maltreatment and psychosis. For instance, one of the most important of these models has shown that childhood maltreatment may result in psychosis via a pathway of posttraumatic stress disorder-related symptoms, including intrusive memories and dissociation [12, 13]. In this conceptual model, “flash-backs could be interpreted as being externally generated, which leads to hallucinatory experiences and hampers reality testing” [12].

The posttraumatic dissociation role in psychosis was already highlighted when Janet [12] characterized hysterical psychosis by its stress-related and dissociative nature. Although dissociation has not been recognized as a diagnostic criterion for any form of psychosis, Moskowitz et al. [14] and Ross [15] (among contemporary authors) suggested that a certain dissociative type of psychosis could potentially respond to trauma-focused psychotherapy. In this regard, accumulative evidence from an updated meta-analysis stated that dissociation—characterized by disruptions to the integrative functioning of several core mental domains— was related to positive psychotic symptoms (especially hallucinations) and less robustly associated with negative psychotic experiences. These findings proposed that certain psychotic symptoms might be better conceptualized as dissociation in nature and support the development of interventions targeting dissociative experiences in treating psychotic symptoms [16].

Although researchers have paid much attention so far to the causal pathways from certain forms of childhood maltreatment to specific psychotic symptoms, they have remained highly unexplained among patients with schizophrenia spectrum and other psychotic disorders [7]. However, a meta-analysis conducted by Alameda et al. [12] showed that dissociative experiences might play a mediating role in the relationship between childhood maltreatment and the development of psychotic symptoms. Nevertheless, several methodological and conceptual problems have been consistently identified in studies on childhood maltreatment, dissociation, and psychosis, which need to be addressed. Firstly, these studies adopted the Dissociative Experiences Scale (DES) to assess dissociation. Although the DES is a well-validated measure of dissociation, its mere use in psychotic patients is controversial due to item content overlap. For instance, item 27 of the DES is designed to get information about voice-hearing experiences directly. Reality distortions and perceptual disturbances are also reflected by eight items within the DES, which might not be able to discriminate between hallucinatory and dissociative experiences [17]. Besides, dissociation appears to be state-dependent among patients with schizophrenia spectrum disorders [18]. Therefore, the diagnostic interviews by applying Structured Clinical Interview for DSM-IV Dissociative Disorders (SCID-D) should be incorporated alongside the DES to avoid measurement artifacts [17, 18]. Given the relatively significant prevalence of undiagnosed dissociative disorders and borderline personality disorder among psychotic patients, the second problem is that these comorbidities emerge when exploring the relationship between psychotic symptoms and dissociation. Hence, it is crucial to evaluate these comorbidities through Structured Clinical Interview for DSM-5 Personality Disorders (SCID-5-PD) and SCID-D [16, 19–21]. Thirdly, it is required to pay attention to different dissociation subtypes. Although recent evidence suggested that absorption may be more associated with psychotic-like experiences, the evidence synthesis based solely on the bivariate relationships between dissociation and psychotic experiences cannot confidently prove whether symptom-specific associations exist between psychotic symptoms and dissociation or between psychotic symptoms and specific dissociative subtypes. In this respect, multivariate analyses might be more practical to answer such questions [16]. Fourthly, it is debated whether neglect and abuse constructs can be statistically differentiated in psychosis. If separate neglect and childhood abuse constructs are validated, studies should apply statistical procedures (e.g., hierarchical regression) so that the effect of abuse is partialled out from neglect impact, and vice versa [22]. Finally, investigations might need to address the measurement of any differences in strength of association between childhood maltreatment and delusions/hallucinations across the psychosis continuum from delusions/hallucinations in nonclinical samples through first-episode psychosis to chronic disorder [7].

Our goal here is to more precisely investigate the causal pathways from childhood maltreatment to psychotic symptoms among psychotic patients by overcoming the methodological problems discussed above. In this respect, we replicated the study by Holowka et al. [23] on childhood maltreatment and dissociation among chronic psychotic patients, while extending the design by including first-episode psychotic patients, and community controls. Additionally, we hypothesized that, in patients with schizophrenia spectrum and other psychotic disorders, the particular forms of dissociation may mediate the association between five major types of childhood abuse and psychotic symptoms, by controlling for gender [8, 24].

## Methods

### Participants

This cross-sectional correlation study was performed from February to December 2019. The G*Power software version 3.1.9.4 was used to compute the sample size of the study. The sample size for 90% power (considering α error probability of 0.05, a medium effect size of 0.15, and 8 predictor variables) was estimated at 136 people [25], which increased to 150 participants regarding a 10% risk of attrition. Finally, a total of 140 individuals with schizophrenia spectrum and other psychotic disorders (i.e., patients diagnosed with schizophrenia, schizophreniform disorder, schizoaffective disorder, delusional disorder, and other specified schizophrenia spectrum and psychotic disorders), including 70 first-episode psychotic patients and 70 chronic psychotic patients, were selected by the systematic random sampling method (with the sampling interval of 3) from the inpatients and outpatients referring to Baharan psychiatric hospital, Zahedan, Iran and were matched based on age, gender, and education level. The inclusion criteria for psychotic patients were: (i) individuals with the diagnoses of schizophrenia spectrum and other psychotic disorders confirmed by an expert psychiatrist via Structured Clinical Interviews for DSM-5: Clinician Version (SCID-5-CV) [26]; (ii) minimum and maximum ages of 18 and 65, respectively; (iii) comprehension ability. We excluded psychotic patients presented with (i) intellectual disability (i.e., intelligence quotient (IQ) score of about 70 or below based on the Wechsler Adult Intelligence Scale-Revised (WAIS-R) [27]), as well as difficulties in conceptual, social, and practical areas of living [26]; (ii) acute physical disease needing emergency interventions; (iii) dementia or other severe brain injuries; (iv) hearing loss and verbal disability; (v) substance-induced psychotic disorder and/or substance use in the last month (according to “chemical dissociation” hypothesis) [28]; (vi) psychotic disorder due to another medical condition; (vii) those who had filled the questionnaires incompletely. Moreover, 70 age-, gender-, and education level-matched community controls were recruited from hospital staff and their relatives and friends. Eligibility criteria for community controls included a score of < 22 in the 28-item General Health Questionnaire (GHQ-28) [29], with confirmed mental health by an expert psychiatrist using SCID-5-CV [26]. The demographic information of participants is listed in Table 1 (*N* = 210).

**Table 1:** Demographic information among the three study groups (*N* = 210)

Demographic data	Categories	1 (*n* = 70)	2 (*n* = 70)	3 (*n* = 70)	Test^a^
		n (%)	n (%)	n (%)	
Age	20–29	2 (2.9)	4 (5.7)	3 (4.3)	χ^2^ (2) = 0.55
	30–39	13 (18.6)	11 (15.7)	8 (11.4)	
	40–49	28 (40.0)	30 (42.9)	32 (45.7)	
	50–59	15 (21.4)	18 (25.7)	18 (25.7)	
	60–70	12 (17.1)	7 (10.0)	9 (12.9)	
Gender	Male	44 (62.9)	46 (65.7)	44 (62.9)	χ^2^ (2) = 0.16
	Female	26 (37.1)	24 (34.3)	26 (37.1)	
Education level	Illiterate	4 (5.7)	3 (4.3)	2 (2.9)	χ^2^ (2) = 0.43
	non-degree	44 (62.9)	47 (67.1)	45 (64.3)	
	high school diploma	19 (27.1)	17 (24.3)	19 (27.1)	
	Academic degree	3 (4.3)	3 (4.3)	4 (5.7)	

*Note*: ^1^First-episode psychotic patients; ^2^Chronic psychotic patients; ^3^Community controls

*Note*: ^a^Statistical analyzing applied chi-square test and Kruskal-Wallis test

^*^p < 0.05; ^**^p < 0.01; ^***^p < 0.001

### Procedures

After approval of the research project by the Zahedan University of Medical Sciences Research Ethics Committee (REC) Reg. no. IR.ZAUMS.REC.1398.413, all of the patients and community controls were informed about the nature and objectives of the study. Also, if they agreed to participate, they were led to a private room by the expert psychiatrist to complete the assessments. After obtaining the consent from the participants, the patients with schizophrenia or other psychotic disorders were divided into first-episode psychotic patients and chronic psychotic patients based on the fact that they experienced the first episode of psychosis or had been experiencing it for more than 2 years, respectively [30]. Next, all participants of the three groups were assessed using DES [31] and Childhood Trauma Questionnaire-Short Form (CTQ-SF) [32] with the help of researchers. In contrast, Positive and Negative Syndrome Scale (PANSS) [33] was only performed to evaluate patients with schizophrenia and other psychotic disorders by an expert psychiatrist. Moreover, the patients with schizophrenia spectrum and other psychotic disorders were assessed via a SCID-5-PD [34] and SCID-D [35] in terms of comorbidity with borderline personality disorder and dissociative disorders. To abide by the declaration of Helsinki [36], the individuals were told that their participation was voluntary and they could leave the study for any reason. All participants were also assured of the confidentiality of their personal data and answers.

### Instruments

#### CTQ-SF

The Persian version of CTQ-SF was used to investigate childhood maltreatment. This 28-item questionnaire includes 5 main components, i.e., emotional abuse, physical abuse, sexual abuse, emotional neglect, and physical neglect, which are scored on a 5-point Likert scale [32]. In Iran, Garrusi and Nakhaee [32] reported the test-retest reliability coefficient of this questionnaire to be 0.90. They also determined the internal consistency reliability coefficients for the four subscales of nonsexual abuse, sexual abuse, emotional neglect, and physical neglect at 0.86, 0.85, 0.84, and 0.6, respectively, with an average of 0.79. In our study, the Cronbach’s alpha coefficient for the five subscales of sexual abuse, emotional abuse, physical abuse, emotional neglect, and physical neglect was 0.88, 0.75, 0.82, 0.69, and 0.64, respectively.

#### DES

The Persian version of DES was employed to assess dissociative symptoms. In this 28-item questionnaire, participants are asked to score their experiences for each element based on a 10-point scale from never (0%) to always (100%) [31]. Sajadi et al. [31] reported the Cronbach’s alpha coefficient of this scale to be 0.92 for Persian cases. Using factor analysis, they also showed three factor structures, namely dissociative amnesia, depersonalization/derealization, and absorption, and scored each of them separately. In our study, the Cronbach’s alpha coefficient was 0.90 for DES total score and 0.85, 0.87, and 0.79 for the subscales of dissociative amnesia, depersonalization/derealization, and absorption, respectively.

#### PANSS

The severity of psychotic symptoms was investigated using the Persian version of PANSS. This is a 30-item scale that includes three subscales of positive symptoms (7 questions), negative symptoms (7 questions), and general psychopathology symptoms (16 questions), which are scored based on a 5-point Likert scale (1 = absent, 2 = minimal, 3 = moderate, 4 = severe, and 5 = extreme). Accordingly, the minimum and maximum scores are 30 and 150, respectively [33]. In Iran, Heshmati [33] estimated the Cronbach’s alpha coefficient of this scale at 0.77, whose validity was confirmed according to the factor analysis results. We reported the Cronbach’s alpha coefficient for the subscales of positive symptoms, negative symptoms, and general psychopathology symptoms to be 0.80, 0.78, and 0.70, respectively.

#### SCID-D

SCID-D was used to evaluate dissociative disorders. This semistructured interview assesses five dimensions of dissociative symptoms, including amnesia, depersonalization, derealization, identity confusion, and identity alternation, on a four-point scale ranging from 0 (none) to 4 (severe) based on patients’ descriptions. In previous studies, SCID-D had good-to-excellent reliability and validity. The Persian version of SCID-D yielded 100% agreement with the diagnosis of dissociative disorders [35]. In the present study, the information and scores obtained from SCID-D were used to identify patients with comorbid dissociative disorders based on DSM-5 criteria.

#### SCID-5-CV

SCID-5-CV is a structured interview for major DSM-5 diagnoses, conducted by an expert psychiatrist familiar with diagnostic criteria and classification of disorders in DSM-5. Numerous studies have reported acceptable reliability and validity of SCID-5-CV [26].

#### SCID-5-PD

This tool is a structured diagnostic interview for clinicians and researchers to assess 10 DSM-5 personality disorders across clusters A, B, and C and other specified personality disorders. The reliability and validity of SCID-5-PD have been found suitable in different studies [34].

#### GHQ-28

This 28-item questionnaire is scored based on a 4-point Likert scale (0–3); its overall scores thus range between 0 and 84. A score below the cut-off point of 22 indicates better mental health of an individual [29]. In Iran, Taghavi [29] reported the Cronbach’s alpha coefficient for the total scale to be 0.93. He also performed the factor analysis and managed to show the four factors of depression, anxiety, social dysfunction, and somatic symptoms. In our research, the Cronbach’s alpha coefficient for the total scale was obtained of 0.84.

#### WAIS-R

IQ was measured via WAIS-R, whose validity and reliability had been investigated in an Iranian study [27]. Subscales of WAIS-R showed the validity of 0.69–0.87 in the test-retest stability. Furthermore, their internal consistency was estimated at 0.77–0.88 using the split-half method. WAIS-R was performed by a trained clinical psychologist.

### Statistical analysis

The data were analyzed by SPSS V25.0 software at the significance level of *p* < 0.05. Statistical analysis was conducted using descriptive statistics, such as mean and standard deviation. Chi-square test was performed to compare demography among the three study groups. Further, the analysis of variance (ANOVA) was utilized to compare the mean scores of CTQ-SF and DES in the three study groups. In ANOVA, the Scheffé test is used for post hoc analysis. Subsequently, in the two groups of first-episode psychotic patients and chronic psychotic patients, the point-biserial correlation coefficient and Pearson correlation coefficient were employed to assess the relationship between gender [8, 24], child maltreatment, dissociation, and the severity of psychotic symptoms. Further, to examine the mediating role of dissociation in the relationship between childhood maltreatment and psychotic symptoms in the two groups of patients, the mediation analysis procedures with bootstrap sampling were performed. According to Preacher and Hayes’ recommendation [37], the bootstrap method estimates direct, indirect, and total effects. All paths were estimated via ordinary least squares regression. These analyses were performed using the Hayes’ PROCESS macro method, a computational procedure for SPSS [37]. As stated by Preacher and Hayes [38], the mediating role exists provided that the indirect effect is significant and the confidence interval excludes a zero value. Only dependent and independent variables that exhibited statistically significant correlations were involved in the mediation analysis.

## Results

### Preliminary analysis

**Fig. 1:**
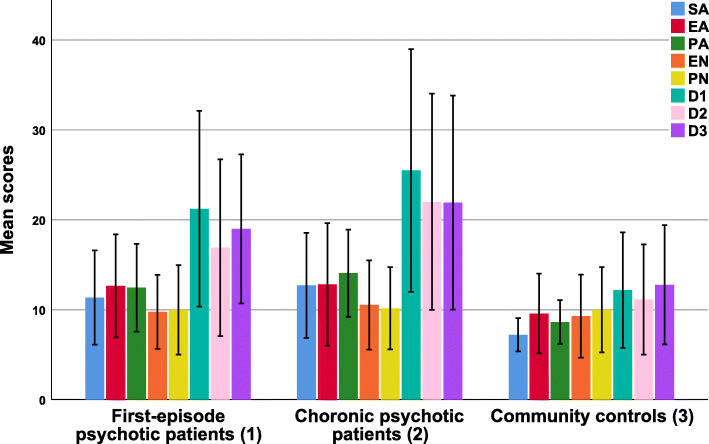
Comparing mean scores of Childhood Trauma Questionnaire-Short Form and Dissociative Experiences Scale among three study groups (*N* = 210). Note. D1: Amnesia; D2: Depersonalization/Derealization; D3: Absorption; EA: Emotional Abuse; EN: Emotional Neglect; PA: Physical Abuse; PN: Physical Neglect; SA: Sexual Abuse. Note. Statistical analyzing applied analysis of variance (ANOVA). SA: F (2, 207) = 26.47^***^; Scheffé post hoc test: 1 & 2 > 3. EA: F (2, 207) = 7.06^**^; Scheffé post hoc test: 1 & 2 > 3. PA: F (2, 207) = 30.71^***^; Scheffé post hoc test: 1 & 2 > 3. EN: F (2, 207) = 1.66. PN: F (2, 207) = 0.13. D1: F (2, 207) = 33.94^***^; Scheffé post hoc test: 2 > 1 > 3. D2: F (2, 207) = 28.49^***^; Scheffé post hoc test: 2 > 1 > 3. D3: F (2, 207) = 21.59^***^; Scheffé post hoc test: 2 > 1 > 3. ^*^p < 0.05; ^**^p < 0.01; ^***^p < 0.001. Note. This clustered bar chart was created by IBM SPSS Statistics V25.0 (www.ibm.com)

The comparison of mean scores of different types of childhood maltreatment in the three study groups showed that psychotic patients (i.e., first-episode psychotic patients and chronic psychotic patients), compared to the community controls, received higher scores in the subscales of sexual abuse, emotional abuse, and physical abuse, which were statistically significant (*p* < 0.001, *p* = 0.001, and *p* < 0.001, respectively). However, no considerable difference in such scores was observed between first-episode psychotic patients and chronic psychotic patients. In addition, ANOVA and post hoc analysis revealed no significant difference in the mean scores of emotional neglect and physical neglect among the three study groups. Mean scores of dissociative experiences showed a significant difference among the three study groups (p < 0.001). In other words, the results obtained from the post hoc analysis implied higher mean scores of dissociative experiences in chronic psychotic patients (see Fig. 1). Moreover, using SCID-5-PD and SCID-D, the interviews demonstrated that four chronic psychotic patients were comorbid with borderline personality disorder (*n* = 3) and dissociative disorders (*n* = 1), and one first-episode psychotic patient was comorbid with dissociative disorders.

Moreover, the results obtained from the correlation matrix demonstrated that sexual abuse had a significant and positive correlation with dissociative amnesia (r = 0.62, *p* < 0.001), depersonalization/derealization (r = 0.67, p < 0.001), absorption (r = 0.64, p < 0.001), positive symptoms (r = 0.42, p < 0.001), negative symptoms (r = 0.29, p < 0.001), and general psychopathology symptoms (r = 0.42, p < 0.001). Physical abuse had also a significant and positive correlation with positive symptoms (r = 0.31, p < 0.001), negative symptoms (r = 0.27, p < 0.001), and general psychopathology symptoms (r = 0.29, p < 0.001). A significant correlation was observed between each subscale of DES and PANSS as well. Nonetheless, no significant correlation was found between gender, other types of childhood maltreatment, and PANSS subscales (see Table 2).

**Table 2:** Correlations of study variables among patients with schizophrenia spectrum and other psychotic disorders (*N* = 140)

Variables	SA	EA	PA	EN	PN	D1	D2	D3	PANSS1	PANSS2	PANSS3	Gender
SA	–											
EA	0.01	–										
PA	0.12	0.09	–									
EN	0.01	0.21^*^	0.08	–								
PN	−0.07	−0.01	−0.11	0.02	–							
D1	0.62^***^	0.00	0.16	−0.02	−0.01	–						
D2	0.67^***^	−0.08	0.07	−0.03	− 0.05	0.57^***^	–					
D3	0.64^***^	0.02	0.10	−0.07	−0.00	0.72^***^	0.68^***^	–				
PANSS1	0.42^***^	0.11	0.31^***^	0.08	−0.05	−0.48^***^	−0.28^**^	0.45^***^	–			
PANSS2	0.29^***^	0.02	0.27^**^	−0.07	0.00	0.26^**^	0.27^**^	−0.33^***^	0.04	–		
PANSS3	0.42^***^	0.15	0.29^***^	0.00	0.04	−0.42^***^	−0.37^***^	0.53^***^	0.49^***^	0.24^**^	–	
Gender	−0.13	0.01	0.04	−0.06	0.06	−0.08	−0.09	− 0.16	−0.13	− 0.12	−0.04	–

*Note: D1* Amnesia, *D2* Depersonalization/Derealization, *D3* Absorption, *EA* Emotional Abuse, *EN* Emotional Neglect, *PA* Physical Abuse, *PANSS1* Positive Symptoms, *PANSS2* Negative Symptoms, *PANSS3* General Psychopathology Symptoms, *PN* Physical Neglect, *SA* Sexual Abuse

^*^p < 0.05; ^**^p < 0.01; ^***^p < 0.001

### Predictors of positive, negative, and general psychopathology symptoms

Table 3 represents a summary of regression analysis results obtained by Hayes’ PROCESS macro method. In model 1, the results showed that sexual abuse (β = 0.32, *p* = 0.046), dissociative amnesia (β = − 0.16, *p* = 0.025), absorption (β = 0.20, *p* = 0.038), and physical abuse (β = 0.41, *p* = 0.001) could explain 33% of the variance of positive symptoms among the patients with schizophrenia spectrum and other psychotic disorders (F (5, 134) = 13.76, p < 0.001). In model 2, only physical abuse (β = 0.25, *p* = 0.003) was able to explain 17% of the variance of negative symptoms (F (5, 134) = 5.73, *p* < 0.001). Also, in model 3, merely absorption (β = 0.44, *p* < 0.001) and physical abuse (β = 0.48, *p* < 0.001) could explain 35% of the variance of general psychopathology symptoms (F (5, 134) = 14.79, *p* < 0.001).

**Table 3:** Model summary of multiple regression analysis to evaluate the predictor variables of psychotic symptoms among patients with schizophrenia spectrum and other psychotic disorders (*N* = 140)

Model 1 (Response variable: PANSS1): R = 0.58; R^2^ = 0.33; F (5, 134) = 13.76^***^					
Explanatory variables	β	SE	t	LLCI	ULCI
SA	0.32	0.16	2.00^*^	0.004	0.646
D1	−0.16	0.07	−2.25^*^	−0.318	− 0.021
D2	−0.14	0.08	− 1.75	− 0.303	0.018
D3	0.20	0.09	2.09^*^	0.011	0.400
PA	0.41	0.12	3.29^**^	0.165	0.662
Model 2 (Response variable: PANSS2): R = 0.42; R^2^ = 0.17; F (5, 134) = 5.73^***^					
Explanatory variables	β	SE	t	LLCI	ULCI
SA	0.11	0.11	1.04	−0.103	0.332
D1	0.02	0.05	0.41	−0.079	0.122
D2	0.01	0.05	0.29	−0.092	0.125
D3	−0.12	0.06	−1.84	−0.255	0.008
PA	0.25	0.08	2.96^**^	0.083	0.421
Model 3 (Response variable: PANSS3): R = 0.59; R^2^ = 0.35; F (5, 134) = 14.79^***^					
Explanatory variables	β	SE	t	LLCI	ULCI
SA	0.23	0.18	1.25	−0.133	0.598
D1	−0.00	0.08	−0.00	− 0.170	0.169
D2	−0.03	0.09	−0.37	−0.218	0.149
D3	0.44	0.11	3.92^***^	0.218	0.662
PA	0.48	0.14	3.39^***^	0.203	0.770

*Note*: *D1* Amnesia, *D2* Depersonalization/Derealization, *D3* Absorption, *LLCI* Lower Limit of Confidence Interval, *PA* Physical Abuse, *PANSS1* Positive Symptoms, *PANSS2* Negative Symptoms, *PANSS3* General Psychopathology Symptoms, *SA* Sexual Abuse, *ULCI* Upper Limit of Confidence Interval

^*^p < 0.05; ^**^p < 0.01; ^***^p < 0.001

### Mediation analysis

**Fig. 2:**
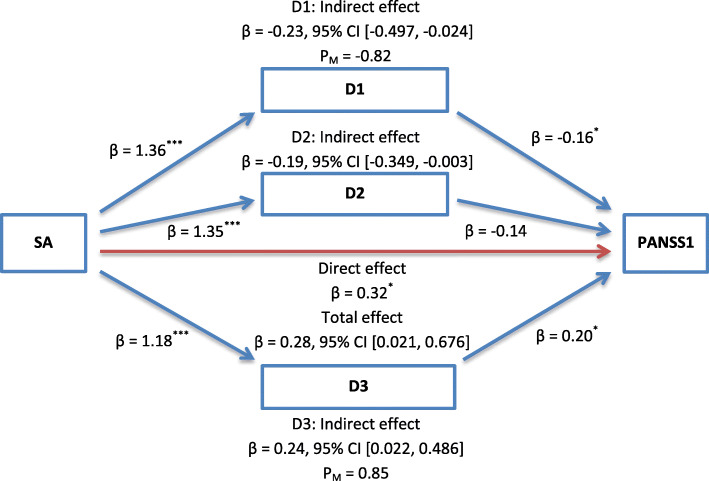
Illustration of the results of the mediation analysis described in the text, which tested dissociative amnesia, depersonalization/derealization, and absorption as the potential mediators of the relationship between sexual abuse and positive symptoms by controlling for physical abuse among patients with schizophrenia spectrum and other psychotic disorders (*n* = 140). Note. D1: Amnesia; D2: Depersonalization/Derealization; D3: Absorption; PANSS1: Positive Symptoms; SA: Sexual Abuse. P_M_: Effect size (ratio of indirect to total effect). ^*^p < 0.05; ^**^p < 0.01; ^***^p < 0.001

Mediation analysis was performed in SPSS using Hayes’ process tool (model = 4, bootstrap samples = 5000). As already assumed, a significant indirect effect of sexual abuse was distinctly observed on positive symptoms through dissociative amnesia and absorption (β = − 0.23, 95% confidence interval (CI): − 0.497, − 0.024; β = 0.24, 95% CI: 0.022, 0.486, respectively). The mediators (i.e., amnesia and absorption) could account for roughly 82 and 85% of the total effect of sexual abuse on positive symptoms, respectively. Hence, the overall hypothesis that dissociative amnesia and absorption mediated the effect of sexual abuse on positive symptoms was supported (see Fig. 2).

## Discussion

To the best of our knowledge, this is the first study on the relationship between childhood maltreatment, dissociative experiences, and psychotic symptoms in Iranian patients with schizophrenia spectrum and other psychotic disorders. The findings of the present study can be divided into six major parts. As the first part, the results of this study indicated that only the mean scores of sexual abuse, emotional abuse, and physical abuse (not mean scores of emotional neglect and physical neglect) were significantly higher in psychotic patients than the community controls. Despite that, no significant difference was observed between first-episode psychotic patients and chronic psychotic patients. These findings were consistent with a priori hypothesis of the association between childhood abuse and psychotic symptoms, proposed in previous studies [39–41]. For example, Daalman et al. [41] observed higher rates of childhood sexual and emotional abuse in both groups of hallucinated objects, irrespective of their disease status. These findings have introduced childhood maltreatment as a specific risk factor in the development of psychotic symptoms.

As the second main finding of the present study, the mean scores of dissociative experiences in the three study groups were significantly different, and the results obtained from the post hoc analysis confirmed higher mean scores of dissociative experiences in chronic psychotic patients. Previously, Braehler et al. [42] investigated first-episode psychotic patients, chronic psychotic patients, and community controls, realizing that chronic psychotic patients experienced higher levels of dissociative symptoms. The greater dissociation in chronic psychotic patients might be attributed to traumatic experiences after childhood; the issue disregarded in the present study. Psychiatric patients, compared to the general population, are more likely to experience additional traumatic events (e.g., assault) in adulthood [43]. Meanwhile, chronic patients might be at a higher risk of re-traumatization due to more coercive admissions and hospitalization [44]. Accordingly, if dissociative symptoms are considered the result of a set of childhood and adulthood traumatic events, chronic psychotic patients are expected to be at a greater risk of more severe dissociation due to experiencing multiple traumas [42]. Nevertheless, further studies are required to investigate potential cumulative effects of adulthood trauma on dissociation. Another probable reason for higher levels of dissociation in the patients with schizophrenia spectrum and other psychotic disorders (particularly chronic psychotic patients) is the etiological and phenomenological overlap between dissociative and psychotic symptoms [19]. In this regard, previous studies identified a subgroup of schizophrenic patients with high levels of childhood maltreatment and dissociation who met diagnostic criteria for borderline personality disorder or dissociative disorders [20]. Childhood adversities have been shown to be higher in schizophrenia patients with comorbid borderline personality disorder than those without borderline personality disorder [21]. Nonetheless, in our study, only four chronic psychotic patients (including 3 patients with comorbid borderline personality disorder and one patient with comorbid dissociative disorders), and one first-episode psychotic patient (with comorbid borderline personality disorder) made this explanation improbable. Finally, in recent years, it has been suggested that schizophrenia is best understood as a disorder of consciousness and self-experience (disturbed ipseity) that involved two key aspects of “hyper-reflexivity” (i.e., forms of exaggerating and alienating self-consciousness) and “diminished self-affection” (i.e., a diminished sense of existing as a subject of awareness or agent of action) [45]. This hypothesis may explain the apparent heterogeneity of psychotic symptoms among patients with schizophrenia spectrum and other psychotic disorders.

As the third main finding of the present study, there was no association between gender, child maltreatment, dissociation, and the severity of psychotic symptoms among patients with schizophrenia spectrum and other psychotic disorders. However, a recent review article revealed that women might be at a greater risk of sexual abuse than men [24]. This may be explained by cultural norms (such as shame, taboos and modesty, virginity, status of females, etc.) affecting the likelihood of whether child sexual abuse is discovered by an adult or disclosed by the child. For example, where cultural norms favor males over females, a girl’s report of sexual abuse by a boy or a man may be discounted [46].

As the fourth main result of the present study, the summary of regression analysis results revealed that for the patients with schizophrenia spectrum and other psychotic disorders, positive symptoms were related to sexual abuse, dissociative amnesia, absorption, and physical abuse, negative symptoms were associated with physical abuse, and general psychopathology symptoms were related to absorption and physical abuse. These findings were consistent with studies conducted by Sheffield et al. [39], Bendall et al. [47], and Read et al. [48], whereas they were inconsistent with results obtained by Bell et al. [49]. However, contrary to Sheffield et al. [39], we observed no relationship between emotional abuse and psychotic symptoms. This suggests that further studies are required to decide whether emotional abuse can account for psychotic symptoms or not. In addition, our study illustrated that physical abuse could be associated with psychotic symptoms even in the absence of sexual abuse and dissociation as well. This finding argues that some of the psychotic patients might have adapted differently to childhood adversities. In this respect, observing a relationship between childhood maltreatment and negative symptoms, Vogel et al. [50] concluded that negative symptoms (i.e., a constant state of down-regulation of emotion and social engagement) could be an alternative adaptive response to childhood adversities.

As the fifth finding of this study, dissociation mediated the relationship between sexual abuse and positive symptoms, which agreed with the similar results in clinical groups and preliminary research with nonclinical participants [51–54]. Overall, this finding supports the information-processing theory proposed by Holmes et al. [55] who argued that peri-traumatic dissociation resulted in poorly encoded autobiographical representations by disruption of information processing, which might be later re-experienced as traumatic intrusions (e.g., hallucinations). Further, recent studies have suggested that weakened cognitive inhibition might represent the prevailing cognitive concomitant of dissociation [56]. In line with this theory, recent experimental evidence has highlighted the importance of inhibitory processes in the explanation of auditory hallucinations [57, 58]. Nevertheless, further research is required to determine whether such processes are able to explain the observed relationship between dissociation and positive symptoms.

As another interesting finding of our study, the regression analysis revealed that dissociative symptoms were related to psychotic symptoms in some cases independent of exposure to childhood maltreatment, suggesting that dissociation did not develop as a result of childhood adversities in some patients. These findings led to the following question: “Do various psychotic symptoms have distinct etiologies?” In answer to this question, recent evidence has suggested the presence of substantial overlaps in socio-environmental and biological risks across specific symptoms and diagnostic categories; accordingly, a move has been taken toward transdiagnostic therapies [59–62]. Nevertheless, many risk and resilience factors might exist for each symptom, and the relative significance of each factor is likely to differ from person to person. This highlights the necessity to create individualized formulations to better understand the development of distressing symptoms from a psychological therapy perspective [16].

As the latest finding of this study, the results revealed that absorption mediated the relationship between sexual abuse and positive symptoms, agreeing with the results obtained by Cole et al. [51] and Perona-Garcelán et al. [54]. Absorption is a form of intensively focused attention wherein an individual becomes immersed in their mental imagery so that these events seem to happen in reality, just like what takes place in a hallucinatory experience [51]. In addition, the confusion between reality and imagination due to the disability to determine the veracity of memories can also lead to a fixed, false, and idiosyncratic belief that is perceived as delusion [51, 54]. We also found out that dissociative amnesia had a mediating role in the relationship between sexual abuse and positive symptoms. Previously, Kennerley [63] following Holmes et al. [55], emphasized distinct functions of different types of dissociation. Indeed, tuning in (absorption) can cause the re-living of intrusive peri-traumatic information in the forms of flash-backs and hallucinations. Tuning out (dissociative amnesia) might make a person unable to access traumatic information in the memory [51]. Accordingly, absorption and dissociative amnesia are expected to have positive and negative mediating roles, respectively, exactly consistent with what was observed in our study. An unexpected finding was about the lack of mediating role of depersonalization/derealization in the relationship between sexual abuse and positive symptoms; consistent with the study by Cole et al. [51] and inconsistent with the results obtained by Perona-Garcelán et al. [53, 54]. This might be due to negligible detrimental effects of non-pathological depersonalization/derealization on processing information related to adverse events in clinical groups [53].

The present study suffered from some methodological limitations. First, the findings could not be generalized to various cases since the sample size was small and participants were selected from a single geographic region. Second, cross-sectional studies mostly fail to specify a definite reason behind a correlation. This restriction might avoid a deep understanding of the essence of the causal relationship between childhood maltreatment, dissociative experiences, and psychotic symptoms. As the third limitation, this study used self-report scales that can only identify the emotions of patients through the assessment and are not able to reflect their real emotions. Hence, it is suggested that future studies should focus on methodological limitations, such as sole reliance on self-report scales due to memory bias and demand characteristics, lack of empirical data, and disregarding ethnic differences. It is worth noting that side effects of antipsychotics (e.g., memory problems, affective flattening, and detachment) may also be mistaken for dissociation during the evaluation of psychotic patients; thus, they should be considered in future studies [16].

Despite the above limitations, our study improved psychopathological comprehension of psychotic symptoms in patients with schizophrenia spectrum and other psychotic disorders. To the best of our knowledge, the present work is the first study that equally assesses and compares the effect of five major types of childhood abuse on various types of psychotic symptoms in Iranian patients with schizophrenia spectrum and other psychotic disorders. This systematic evaluation provided a better opportunity to observe childhood maltreatment as a risk factor in psychosis and allowed us to find specific relationships between sexual abuse, dissociative experiences, and positive symptoms. Understanding such internalized representations can be essential to develop therapeutic interventions and preventive approaches in victims of child maltreatment [64].

## Conclusions

The present study aimed to assess the mediating role of dissociation in the relationship between childhood maltreatment and psychotic symptoms among patients with schizophrenia spectrum and other psychotic disorders. The study results showed that the mean scores of sexual abuse, emotional abuse, physical abuse, and dissociative experiences were higher in psychotic patients (particularly chronic psychotic patients) than community controls. However, in the three study groups, no significant gender-based difference was observed between the mean scores of dissociative experiences and various types of childhood maltreatment. In addition to confirming a relationship between physical abuse and psychotic symptoms (even in the absence of sexual abuse), this study implied the mediating role of absorption and dissociative amnesia in the relationship between sexual abuse and positive symptoms. These findings emphasized the importance of assessing the history of childhood maltreatment (particularly sexual and physical abuse history) and dissociative experiences in psychotic patients (regardless of their gender) to ensure the proposal of the most suitable and effective therapeutic interventions for this group of patients. Nonetheless, further studies using prospective or longitudinal designs are required to better understand the differential contribution of various forms of childhood maltreatment and their potential interactions.

## Data Availability

The datasets generated and analyzed during the current study are not publicly available because no consent was obtained from the participants in this regard. However, the data are available from the corresponding author on a reasonable request.

## Abbreviations

ANOVA: Analysis of variance
CTQ-SF: Childhood Trauma Questionnaire-Short Form
DES: Dissociative Experiences Scale
GHQ-28: The 28-item General Health Questionnaire
IQ: Intelligence quotient
PANSS: Positive and Negative Syndrome Scale
SCID-D: Structured Clinical Interview for DSM-IV Dissociative Disorders
SCID-5-CV: Structured Clinical Interviews for DSM-5: Clinician Version
SCID-5-PD: Structured Clinical Interview for DSM-5 Personality Disorders
WAIS-R: Wechsler Adult Intelligence Scale-Revised
